# Supplementary material for: Splice-Junction-Based Mapping of Alternative Isoforms in the Human Proteome
Source: Cell Rep. Author manuscript; Available in PMC 2020 Jan 15. (PMC6961840; doi:10.1016/j.celrep.2019.11.026)
Supplement: 2 [file NIHMS1546469-supplement-2.zip › DF1/PXD010154/Heart/Heart_59_NEXN_QEMGEEDDVDVRPAR.pdf]

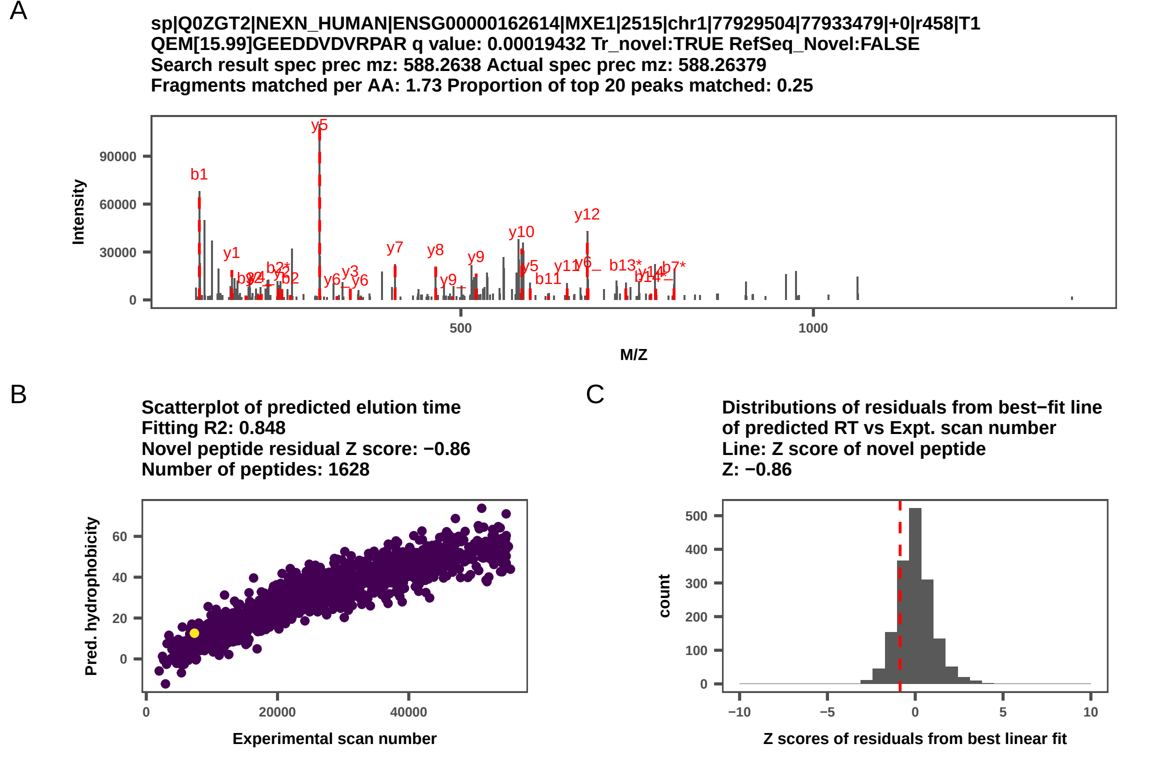

A
sp|Q0ZGT2|NEXN_HUMAN|ENSG00000162614|MXE1|2515|chr1|77929504|77933479|+0|r458|T1
QEM[15.99]GEEDDVDVRPAR q value: 0.00019432 Tr_novel:TRUE RefSeq_Novel:FALSE
Search result spec prec mz: 588.2638 Actual spec prec mz: 588.26379
Fragments matched per AA: 1.73 Proportion of top 20 peaks matched: 0.25
Intensity
90000
60000
30000
0
b1
y5
y1
y7
y8
y9
y10
y12
y3
y6
b11
b13*
b7*
500
1000
M/Z
B
Scatterplot of predicted elution time
Fitting R2: 0.848
Novel peptide residual Z score: −0.86
Number of peptides: 1628
Pred. hydrophobicity
60
40
20
0
20000
40000
Experimental scan number
C
Distributions of residuals from best−fit line
of predicted RT vs Expt. scan number
Line: Z score of novel peptide
Z: −0.86
count
500
400
300
200
100
0
−10
−5
0
5
10
Z scores of residuals from best linear fit
